# Sanguinarine Attenuates Neuropathic Pain in a Rat Model of Chronic Constriction Injury

**DOI:** 10.1155/2021/3689829

**Published:** 2021-04-21

**Authors:** Ping Li, Yan-Xiu Wang, Guang Yang, Zun-Cheng Zheng, Chao Yu

**Affiliations:** ^1^Department of Physical Medicine and Rehabilitation, Taian City Central Hospital, Taian, Shandong, China; ^2^Department of Pain Medicine, Taian City Central Hospital, Taian, Shandong, China

## Abstract

**Objective:**

There is still no effective treatment of neuropathic pain. Sanguinarine is a natural plant medicine with anti-inflammatory effects, but its effect on neuropathic pain remains unclear. This study was aimed at investigating the potential of sanguinarine to attenuate neuropathic pain.

**Methods:**

Neuropathic pain was induced by chronic constriction injury (CCI) of the sciatic nerve. Rats were randomly divided into several groups: sham, CCI, CCI+SG (1.00 mg/kg), CCI+SG (2.50 mg/kg), and CCI+SG (6.25 mg/kg). SG was injected intraperitoneally from the day of surgery every three days. The mechanical withdrawal threshold (MWT) and thermal withdrawal latency (TWL) were recorded before surgery and on days 1, 3, 7, and 14 after surgery. The microglia in the spinal dorsal horn were examined by immunofluorescence. p38 MAPK expression in the spinal dorsal horn was detected by PCR and Western blot analysis. Cytokine levels in the spinal dorsal horn were measured by ELISA.

**Results:**

MWT and TWL were significantly reduced in the CCI group, but sanguinarine recovered MWT and TWL in the CCI group. In addition, sanguinarine inhibited the activation of microglia and decreased the expression of p-p38 and TNF-*α*, IL-1*β*, and IL-6 in the spinal dorsal horn of the CCI group in a dose-dependent manner.

**Conclusions:**

Our results suggest that sanguinarine can attenuate neuropathic pain via inhibiting the activation of microglia and the activation of the p38 MAPK signaling pathway.

## 1. Introduction

Neuropathic pain is a common type of chronic pain, and about one-fifth of patients with chronic pain have neuropathic pain [1]. Neuropathic pain is caused by the damage to the sensory nervous system of the body or lesions [2]. Due to the complicated pathogenesis and the lack of effective treatment methods, neuropathic pain has become a worldwide public health problem [2]. An epidemiological study showed that neuropathic pain afflicted 9% of adults to different levels and increased the incidence of anxiety, depression, and other diseases [3, 4]. Therefore, new drugs with better therapeutic effects and less side effects are urgently needed to treat neuropathic pain.

Sanguinarine (SG) is an alkaloid extracted from the root of Sanguinaria canadensis and other poppy *Fumaria* species with antibacterial, antiviral, anti-inflammatory, antioxidant, and antitumor effects [5–7]. Recent studies have shown that SG could inhibit the occurrence and development of inflammation [8–10]. However, the effect of SG on neuropathic pain remains unclear.

The aim of this study was to establish an animal model of neuropathic pain induced by CCI of the sciatic nerve and evaluate the effects of SG on neuropathic pain. Meanwhile, the activation of microglia, phosphorylation of p38, and the expression levels of TNF-*α*, IL-1*β*, and IL-6 in the spinal dorsal horn were investigated to expose the possible mechanisms of the action of SG on neuropathic pain.

## 2. Materials and Methods

### 2.1. Animals

All experiments were approved by the Animal Ethics Committee of Shandong University and performed in strict accordance with the National Guidelines for Animal Use and Care. Adult male Sprague-Dawley rats (weight 180-200 g) were provided by the Shandong Animal Experimental Animal Center (animal license number SCXK 2012-0001) and kept in a humidity control room at 24 ± 1°C with a light/dark cycle and free access to food and water. SG was purchased from Pufei De Biotech Co., Ltd. (Chengdu, China). Rats were randomly divided into 5 groups (12 rats in each group): sham group, CCI group, CCI+SG (1.00 mg/kg) group, CCI+SG (2.50 mg/kg) group, and CCI+SG (6.25 mg/kg) group. CCI was induced in the sciatic nerve of the rats as described previously [11]. In the sham group, the sciatic nerve was exposed but not ligated. On the day of surgery and postoperatively, rats were injected intraperitoneally with SG (1.00, 2.50, and 6.25 mg/kg, once every three days) until the 14th day after surgery. The sham group and the CCI group were administered with the same dose of 0.9% saline.

### 2.2. Behavioral Assessment of Mechanical Sensitivity and Heat Hypersensitivity

The mechanical withdrawal threshold (MWT) was measured using the method of Vivancos et al. [12]. The rats were placed in an inverted transparent Plexiglas cage, placed on a 3 mm thick glass plate, and acclimated for 30 minutes prior to testing. Animals were placed on an elevated wire mesh, and the hind paw was vertically stimulated by the metal probe, and the stimulation intensity gradually increased, resulting in rapid paw contraction or paw licking within 3 seconds. The minimum stimulation intensity that will cause the rat to produce a contraction response was defined as MWT. Each rat was measured three times, at least 5 min intervals, and the mean of the three measurements was regarded as MWT.

The thermal withdrawal latency (TWL) was measured using the method of Hargreaves et al. [13]. Rats was allowed to acclimate for 30 minutes before the test; then, each rat was placed on an elevated glass platform and the heat source was located in the middle of the plantar of the left hind paw under the glass plate. The intensity of thermal stimulation was adjusted to reach 50°C, and the cut-off delay was set to 25 s in order to prevent tissue injury. The time from the start of stimulation to the time the hind paw was lifted, licked, or avoided was recorded as TWL. Each rat was measured three times, at least 5 min intervals, and the mean of the three measurements was regarded as TWL. The behavioral tests were carried out before CCI surgery and 1, 3, 7, and 14 days after CCI.

### 2.3. Immunohistochemical Staining

L4-L6 spinal cord tissue was collected and embedded and then cut transversely into 10 *μ*m thick sections in a freezing microtome (Leica CM1520, Germany). Spinal cord sections were rinsed in phosphate buffer saline (PBS) twice and then incubated in 10% normal goat serum for 1 h. Next, the tissue sections were incubated with a rabbit anti-Iba-1 polyclonal antibody (1 : 1000, ab178847, Abcam) overnight at 4°C and then incubated with goat anti-rabbit IgG (1 : 200, SF134, Solarbio) for 1 h at 37°C. Finally, the sections were mounted with DAPI (Southern Biotech, cat. no. 0100-20) and observed under a confocal microscope (Olympus BX51, Japan).

### 2.4. Western Blot Analysis

Spinal cord tissues were homogenized in RIPA lysis buffer (Cat#R0010, Solarbio) and centrifuged at 13,000 rpm for 15 min at 4°C. The supernatants were collected, and the protein concentration in the lysate was evaluated using a BCA Protein Assay Kit (PC0020, Solanum). Equal amounts of total protein were separated by electrophoresis on sodium dodecyl sulfate-polyacrylamide gel electrophoresis (SDS-PAGE) and transferred to a polyvinylidene fluoride membrane (Merk Millipore, USA). The membrane was incubated with 5% skim milk for 2 h at room temperature and incubated with an antibody for p-p38 (1 : 1000, 4511T, Cell Signaling), p38 (1 : 1000, 8690, Cell Signaling), or *β*-actin (1 : 1000, 4970T, Cell Signaling) at 4°C overnight. After washing with TBST, the membrane was incubated with horseradish peroxidase-conjugated goat anti-rabbit IgG. The ChemiDoc MP imaging system (Bio-Rad) was used to detect protein expression, and ImageJ software (version 1.47, USA) was used for densitometry.

### 2.5. Enzyme-Linked Immunosorbent Assay

The spinal cord was collected, and the contents of cytokines TNF-*α*, IL-1*β*, and IL-6 were quantified by rat-specific ELISA kits (MultiSciences, China) according to the manufacturer's instructions.

### 2.6. Statistical Analysis

IBM SPSS Statistics 25.0 software was used for statistical analysis. All data are expressed as mean ± standard error of the mean (SEM) and analyzed by ANOVA followed by the post hoc LSD or Dunnett test. *p* < 0.05 was considered statistically significant.

## 3. Results

### 3.1. SG Recovered Mechanical Allodynia and Thermal Hyperalgesia of CCI Rats

**Figure 1:**
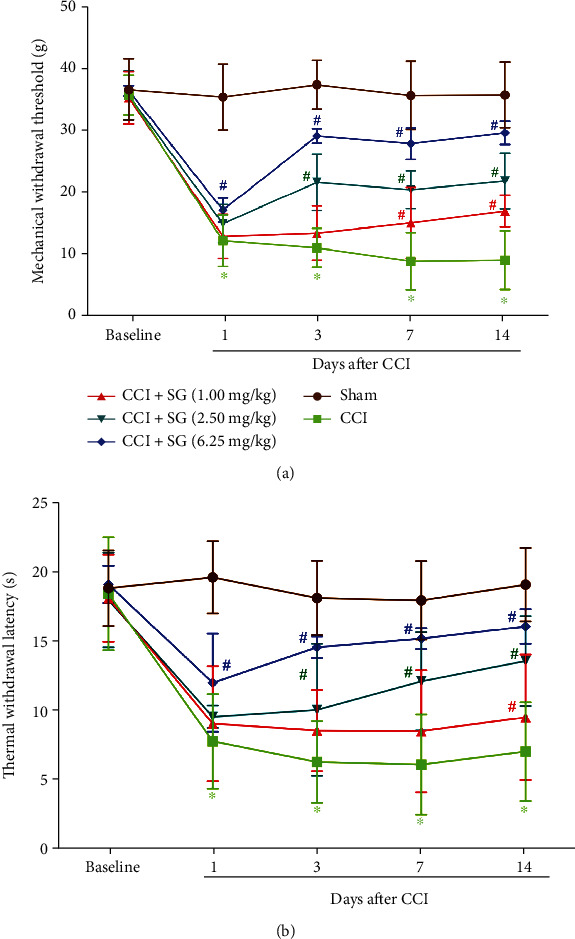
The effects of sanguinarine on mechanical allodynia and thermal hyperalgesia of CCI Rats. (a) MWT in each group. (b) TWL in each group. Data are expressed as mean ± SEM (*n* = 12). ^∗^*p* < 0.05 vs. sham group, ^#^*p* < 0.05 vs. CCI group.

Compared with the sham group, MWT and TWL in the CCI group were significantly lower (Figures 1(a) and 1(b), *p* < 0.05). Compared with the CCI group, the CCI+SG group (1.0, 2.5, and 6.25 mg/kg) recovered CCI-induced reduction in MWT and TWL in a dose-dependent manner (*p* < 0.05). Among them, MVT and TWL of the CCI+SG group (6.25 mg/kg) were significantly increased on the first day after surgery (*p* < 0.05) and lasted for 14 days after surgery. MVT and TWL of the CCI+SG group (2.5 mg/kg) increased significantly from the third day after surgery (*p* < 0.05). Moreover, in the CCI+SG groups (2.5 and 6.25 mg/kg), CCI-induced decrease in MWT and TWL was significantly alleviated from the third day after surgery.

### 3.2. SG Reduced the Levels of Proinflammatory Factors in the Spinal Dorsal Horn of CCI Rats

**Figure 2:**
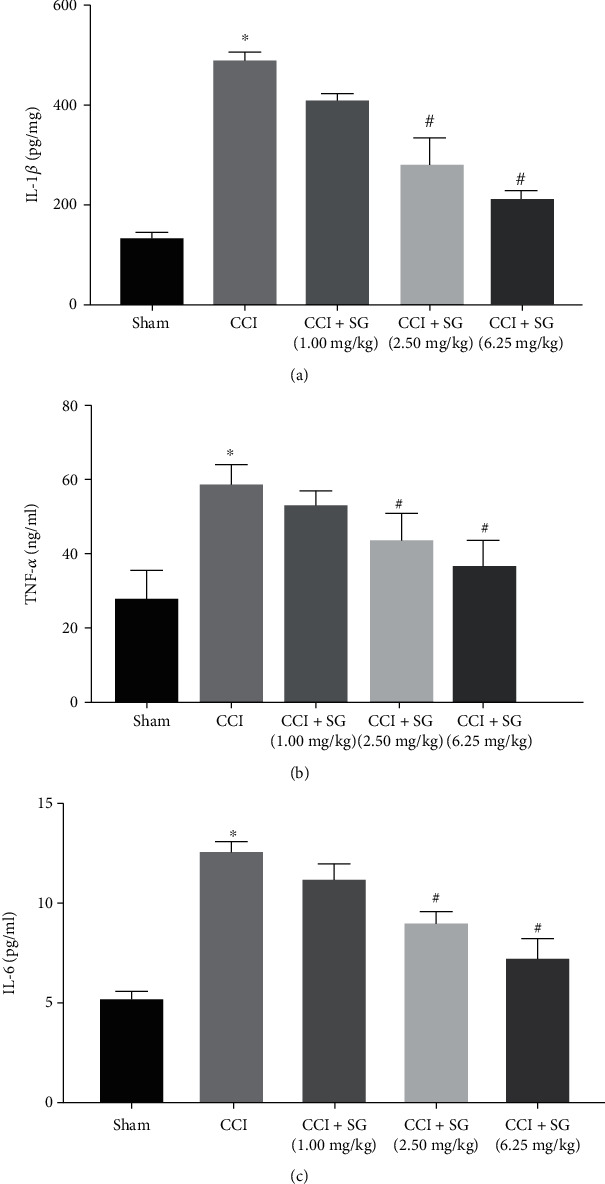
The effects of sanguinarine on IL-1*β*, TNF-*α*, and IL-6 levels in the spinal dorsal horn. (a) IL-1*β* levels. (b) TNF-*α* levels. (c) IL-6 levels. Data are shown as mean ± SEM (*n* = 3). ^∗^*p* < 0.05 vs. sham group, ^#^*p* < 0.05 vs. CCI group.

ELISA results showed that TNF-*α*, IL-1*β*, and IL-6 levels in the spinal cord of the CCI group significantly increased compared with those of the sham group (Figures 2(a)–2(c), *p* < 0.05). Compared with the CCI group, TNF-*α*, IL-1*β*, and IL-6 levels in the spinal cord were slightly decreased in the CCI+SG group (1.0 mg/kg) but had no significant differences (*p* > 0.05). In the CCI+SG group (2.5 mg/kg) and the CCI+SG group (6.25 mg/kg), TNF-*α*, IL-1*β*, and IL-6 levels in the spinal cord were significantly decreased in a dose-dependent manner (*p* < 0.05).

### 3.3. SG Inhibited the Activation of Microglia in the Spinal Dorsal Horn

**Figure 3:**
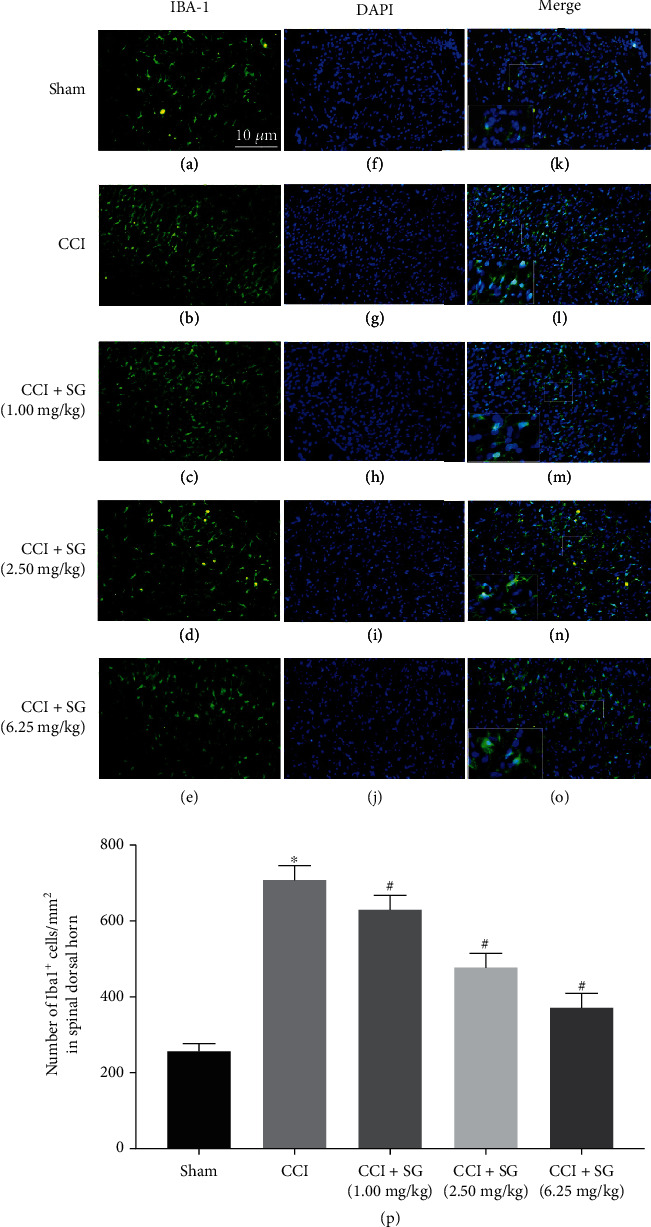
The effects of sanguinarine on microglia in the spinal dorsal horn. (a–e) IHC staining of IBA1-positive microglia in each group. (f–j) DAPI staining of the nuclei of microglia in each group. (k–o) Merging of IBA-1 and DAPI staining. Scale bar = 10 *μ*m. (p) Quantitative analysis of IBA1-positive microglia in each group. Data are shown as mean ± SEM (*n* = 3). ^∗^*p* < 0.05 vs. sham group, ^#^*p* < 0.05 vs. CCI group.

Immunohistochemical staining showed that there were few IBA1-positive microglia in the spinal dorsal horn in the sham group and their cell body was small. However, in the CCI group, the microglia proliferated significantly, with larger cell bodies and thicker branches. Compared with the sham group, the activation of microglia in the CCI group increased significantly. Compared with the CCI group, the activation of microglia in the CCI+SG group (1.0, 2.5, and 6.25 mg/kg) decreased in a dose-dependent manner (Figures 3(a)–3(p)).

### 3.4. SG Inhibited the Activation of p38 in the Spinal Dorsal Horn

**Figure 4:**
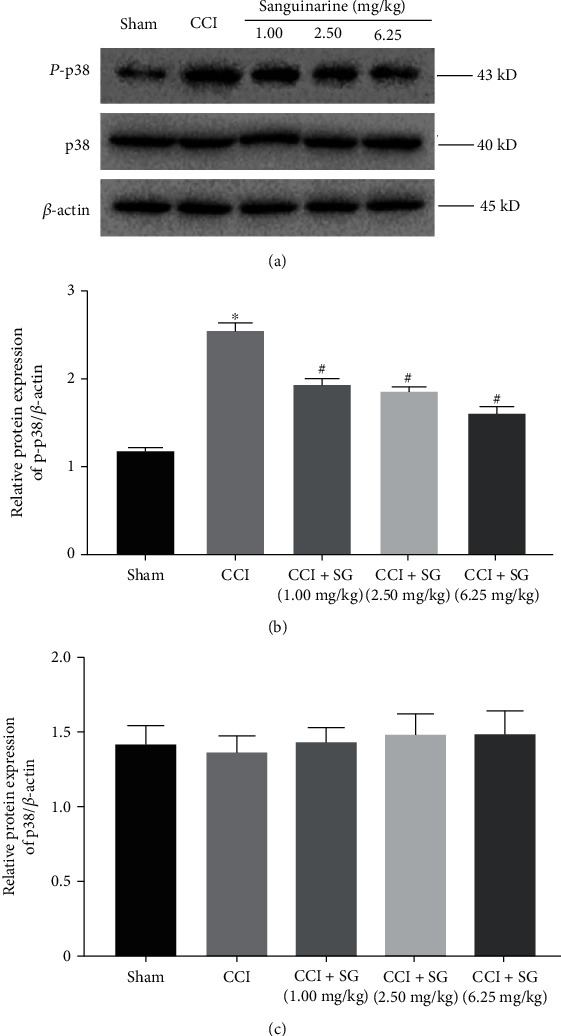
The effects of sanguinarine on the activation of p38 in the spinal dorsal horn. (a) Western blot analysis of p-p38, p38, and *β*-actin protein levels in the spinal cord in each group. (b) Densitometry analysis of p-p38 protein levels in the spinal cord in each group. (c) Densitometry analysis of p38 protein levels in the spinal cord in each group. Data are expressed as mean ± SEM (*n* = 3). ^∗^*p* < 0.05 vs. sham group, ^#^*p* < 0.05 vs. CCI group.

Finally, we investigated the mechanism by which SG relieved neuropathic pain. We performed Western blot analysis to detect p38 and p-p38 protein levels in the spinal dorsal horn of each group (Figure 4(a)). Densitometry analysis showed that activated p38 (p-p38) levels in the spinal dorsal horn of the CCI group significantly increased compared with those of the sham group (Figure 4(b), *p* < 0.05). Compared with the CCI group, p-p38 levels in the spinal dorsal horn of the CCI+SG group (1.0, 2.5, and 6.25 mg/kg) significantly decreased in a dose-dependent manner (*p* < 0.05). However, total p38 protein levels in the spinal dorsal horn of all groups showed no significant change (Figure 4(c)).

## 4. Discussion

Neuropathic pain is a type of chronic pain which is a stubborn pain disease. Because of unclear pathogenesis, most drug treatments for neuropathic pain are ineffective, causing huge economic and mental burden to patients. Many animal models of neuropathic pain have been developed, such as partial sciatic nerve ligation model (PSL) [14], spinal nerve ligation model (SNL) [15], and chronic contractile injury of the sciatic nerve (CCI) [11]. Among them, the CCI model is the most commonly used because it is similar to the characteristics of human neuropathic pain [16]. In this study, we selected the CCI model to induce neuropathic pain in rats. We confirmed that MWT and TWL of the CCI group were significantly lower than those of the sham group, which indicated that our CCI model was successful. Based on this model, we evaluated the effect of SG on neuropathic pain induced by CCI. We found that SG increased MWT and TWL of CCI rats, suggesting that SG has the efficacy to reduce neuropathic pain induced by CCI. It is important to note that while the von Frey model is a good method to measure MWT, in this study, we used the Vivancos model as a classic method to measure MWT which has been widely used in the literature [17].

Next, we explored the possible mechanism responsible for the efficacy of SG on neuropathic pain. It is well known that neuroinflammation plays an important role in pain-related diseases. For example, IL-1*β*, IL-6, and TNF-*α* are closely related to the occurrence and development of pain diseases and cause the amplification of pain [18]. Interestingly, recent studies have shown that SG plays an important role in inhibiting the occurrence and development of inflammation. It is reported that the expression levels of inflammatory factors IL-1*β*, IL-6, and TNF-*α* significantly decreased after SG treatment [8]. Moreover, lipopolysaccharide- (LPS-) induced increase in mRNA expression of IL-1*β*, IL-6, and TNF-*α* could be inhibited by SG treatment [9]. In this study, ELISA showed that IL-1*β*, IL-6, and TNF-*α* were overexpressed in the spinal cord of CCI model rats, but their expression could be downregulated by SG in a dose-dependent manner. Therefore, we speculate that SG may inhibit CCI-induced overexpression of inflammatory factors in the spinal cord.

Previous studies have shown that a large number of inflammatory factors can be released by activated microglia [19]. Microglia are a type of macrophages widely distributed in the central nervous system and play an important role in neuropathic pain [20]. Ionized calcium-binding adaptor molecule-1 (Iba-1) is a special marker of activated microglia [21]. Therefore, in this study, we detected the expression of Iba-1 to determine the activation of microglia. We found that the number of Iba-1-immunopositive microglia cells in the spinal cord of rats increased significantly in the CCI group, the cell body was hypertrophic, and the branches were enlarged, showing a fork-like pattern. However, the number of Iba-1 immunoreactivity microglia in the spinal cord was significantly reduced in a dose-dependent manner after SG treatment, indicating that SG can inhibit the activation of microglia.

Abnormal pain induced by nerve damage depends on the activation of the p38 MAPK signaling pathway [22]. Recent studies suggested that the activation of p38 MAPK signaling is involved in hyperexcitability of dorsal horn neurons and plays a role in the development and maintenance of central sensitization [23]. In this study, we found that the phosphorylation of p38 MAPK was enhanced in the spinal cord of CCI model rats but was attenuated by SG treatment. In addition, the p38 MAPK signaling pathway affects the formation and maintenance of neuropathic pain and central sensitization by regulating the synthesis of proinflammatory cytokines [24]. Therefore, inhibiting the activation of microglia and the activation of p38 MAPK signaling can alleviate hyperalgesia caused by peripheral nerve injury [25]. Our results on the inhibitory effects of SG on inflammatory factors are consistent with the inhibitory effects of SG on the activation of p38 MAPK signaling.

This study has some limitations. First, our results are based on only one animal model of neuropathic pain, and the efficacy of SG should be confirmed on other animal models and even in preclinical investigations. Second, SG is a toxic polycyclic ammonium ion extracted from plants, and its toxicity and side effects should be examined in animal models. Third, we speculated that SG may attenuate neuropathic pain via inhibiting the activation of microglia and the activation of the p38 MAPK signaling pathway. Unfortunately, we checked the literature and found no report on whether SG is able to penetrate the blood-brain barrier. Further studies are needed to examine the penetration of SG into the central nervous system.

In summary, our results suggest that sanguinarine may attenuate neuropathic pain via inhibiting the activation of microglia and the activation of the p38 MAPK signaling pathway, but further clinical studies are needed to confirm our conclusion.

## Data Availability

All data are available from the corresponding author upon request.

## References

[1] Bouhassira D., Lanteri-Minet M., Attal N., Laurent B., Touboul C. (2008). Prevalence of chronic pain with neuropathic characteristics in the general population. *Pain*.

[2] Jensen T. S., Baron R., Haanpaa M. (2011). A new definition of neuropathic pain. *Pain*.

[3] Grace P. M., Hutchinson M. R., Manavis J., Somogyi A. A., Rolan P. E. (2010). A novel animal model of graded neuropathic pain: utility to investigate mechanisms of population heterogeneity. *Journal of Neuroscience Methods*.

[4] Terayama R., Omura S., Fujisawa N., Yamaai T., Ichikawa H., Sugimoto T. (2008). Activation of microglia and p38 mitogen-activated protein kinase in the dorsal column nucleus contributes to tactile allodynia following peripheral nerve injury. *Neuroscience*.

[5] Jeng J. H., Wu H. L., Lin B. R. (2007). Antiplatelet effect of sanguinarine is correlated to calcium mobilization, thromboxane and cAMP production. *Atherosclerosis*.

[6] Montes F. Q., Vázquez-Hernández A., Fenton-Navarro B. (2019). Active compounds of medicinal plants, mechanism for antioxidant and beneficial effects. *Phyton, Int J Exp Botany*.

[7] Ahsan H., Reagan-Shaw S., Breur J., Ahmad N. (2007). Sanguinarine induces apoptosis of human pancreatic carcinoma AsPC-1 and BxPC-3 cells via modulations in Bcl-2 family proteins. *Cancer Letters*.

[8] Wang Q., Dai P., Bao H. (2017). Anti-inflammatory and neuroprotective effects of sanguinarine following cerebral ischemia in rats. *Experimental and Therapeutic Medicine*.

[9] Li W., Li H., Mu Q. (2014). Protective effect of sanguinarine on LPS-induced endotoxic shock in mice and its effect on LPS-induced COX-2 expression and COX-2 associated PGE2 release from peritoneal macrophages. *International Immunopharmacology*.

[10] Niu X., Fan T., Li W., Huang H., Zhang Y., Xing W. (2013). Protective effect of sanguinarine against acetic acid-induced ulcerative colitis in mice. *Toxicology and Applied Pharmacology*.

[11] Bennett G. J., Xie Y. K. (1988). A peripheral mononeuropathy in rat that produces disorders of pain sensation like those seen in man. *Pain*.

[12] Vivancos G. G., Verri W. A., Cunha T. M. (2004). An electronic pressure-meter nociception paw test for rats. *Brazilian Journal of Medical and Biological Research*.

[13] Hargreaves K., Dubner R., Brown F., Flores C., Joris J. (1988). A new and sensitive method for measuring thermal nociception in cutaneous hyperalgesia. *Pain*.

[14] Seltzer Z., Dubner R., Shir Y. (1990). A novel behavioral model of neuropathic pain disorders produced in rats by partial sciatic nerve injury. *Pain*.

[15] Kim S. H., Chung J. M. (1992). An experimental model for peripheral neuropathy produced by segmental spinal nerve ligation in the rat. *Pain*.

[16] Wang L. X., Wang Z. J. (2003). Animal and cellular models of chronic pain. *Advanced Drug Delivery Reviews*.

[17] Li D., Yan Y., Yu L., Duan Y. (2016). Procaine attenuates pain behaviors of neuropathic pain model rats possibly via inhibiting JAK2/STAT3. *Biomol Ther (Seoul)*.

[18] Mazzei L., Ruiz-Roso M. B., De N. (2020). Allicin neuroprotective effect during oxidative/inflammatory injury involves AT1-Hsp70-iNOS counterbalance axis. *Biocell*.

[19] Walter H. L., van der Maten G., Antunes A. R., Wieloch T., Ruscher K. (2015). Treatment with AMD3100 attenuates the microglial response and improves outcome after experimental stroke. *Journal of Neuroinflammation*.

[20] Munoz E. M. (2018). Microglia-precursor cell interactions in health and in pathology. *Biocell*.

[21] Grace P. M., Rolan P. E., Hutchinson M. R. (2011). Peripheral immune contributions to the maintenance of central glial activation underlying neuropathic pain. *Brain, Behavior, and Immunity*.

[22] Ji R. R., Suter M. R. (2007). p38 MAPK, microglial signaling, and neuropathic pain. *Molecular Pain*.

[23] Cao J., Wang J. S., Ren X. H., Zang W. D. (2015). Spinal sample showing p-JNK and P38 associated with the pain signaling transduction of glial cell in neuropathic pain. *Spinal Cord*.

[24] Meotti F. C., Posser T., Missau F. C., Pizzolatti M. G., Leal R. B., Santos A. R. (2007). Involvement of p38MAPK on the antinociceptive action of myricitrin in mice. *Biochemical Pharmacology*.

[25] Zhou C., Shi X., Huang H., Zhu Y., Wu Y. (2014). Montelukast attenuates neuropathic pain through inhibiting p38 mitogen-activated protein kinase and nuclear factor-kappa B in a rat model of chronic constriction injury. *Anesthesia and Analgesia*.

